# Using Prompted Reflective Writing to Demonstrate Learning of Physician Competencies during Global Clinical Rotations

**DOI:** 10.4269/ajtmh.21-0485

**Published:** 2022-03-14

**Authors:** Traci Wells, Pooja Parameshwar, Hendrik Marais, Risa Hoffman, Gitanjli Arora

**Affiliations:** ^1^ David Geffen School of Medicine, University of California, Los Angeles, California;; ^2^ Stanford School of Medicine, Stanford, California;; ^3^ Keck School of Medicine, University of Southern California, Los Angeles, California

## Abstract

Global health education programs have grown in number and rigor with the development of learning objectives, competency frameworks, and assessment tools. This study aimed to assess whether prompted reflective writing could demonstrate medical student learning of physician competencies during global clinical rotations. From 2014 to 2018, 135 medical students who participated in global health clinical rotations responded to four reflective writing prompts. We conducted qualitative content analysis of 487 individual responses using grounded theory and an iterative process to identify themes associated with the eight American Association of Medical College physician competency domains. In response to prompted reflective writing assignments, students demonstrated learning related to all eight competencies. They reflected on systems-based practice while also sharing their growth in knowledge and skills related to personal and professional development, knowledge for practice, interprofessional collaboration, and patient care. In demonstrating practice-based learning and improvement, students additionally reflected on how the experiences during their global clinical rotations might influence their future careers as physicians. Our findings suggest that prompted reflective writing during global clinical rotations allows medical students to demonstrate learning in the competency domains expected of all physician trainees and to reflect on the application of this learning to current and future patient care. In reading students’ writings, we found that prompted reflective writing during global clinical rotations offers an opportunity for students to illustrate the knowledge and skills they have acquired as physicians in training.

## INTRODUCTION

Global health education programs have grown in number and rigor with the development of learning objectives, competency frameworks, and assessment tools. A 2010 survey of medical schools in the United States showed that nearly 90% offered global health programming, with 47% of global health experiences taking place outside of the United States.^1^ In 2020, 22% of all medical students reported having participated in a global health experience while in medical school.^2^ Increasingly, medical schools have developed well-vetted rotations that provide students with predeparture training on ethical engagement embedded within partnerships that strive toward cultural humility, sustainability, capacity building, and bidirectional exchange.^3^^–^^6^ As part of a movement toward more structured and intentional global health education, global health educators have invested in defining competencies in global health.^7^^–^^9^ However, there remains a lack of consensus on the desired global health competencies and how to assess the acquisition of these competencies.^10^^–^^15^ We found that promoting reflective writing during global clinical rotations (GCRs) can provide a means to illustrate medical student learning consistent with the eight American Association of Medical College (AAMC) physician competency domains.

In 2010–2011, the David Geffen School of Medicine at University of California, Los Angeles (UCLA DGSOM) created what is currently known as the Global Health Program (GHP) to provide global health opportunities to medical students. Students in their final year of medical school at DGSOM are able to participate in 3-week global clinical rotations (GCRs) at partner sites in China, Ghana, India, Malawi, Mozambique, Peru, South Africa, and Thailand. Up to one-quarter of students in their final year of medical school have participated in GCRs since 2011. They receive funding, predeparture training,^6^ teaching by host-site faculty, and debriefing upon return.

Between 2011 and 2013, UCLA DGSOM student performance during GCRs was gleaned from clinical rotation evaluation forms completed by host site faculty. Starting in 2014, UCLA DGSOM students have also been assigned reflective writing (RW) prompts via a password-protected online blog site. Only students participating in the rotations and DGSOM faculty have access to the written reflections, with all participants able to respond. To reduce burden to host site faculty and recognizing that students on a short-term rotation may not feel comfortable reflecting openly and in real-time with the host faculty supervising them, host-site faculty and DGSOM faculty supervising the students on-site have not been invited to join the blog. RW has been shown to allow for deeper learning, including improved clinical reasoning, clinical competence, and professional development.^16^^–^^22^ By engaging in RW through online blogging with faculty and peers, support and feedback from faculty and peers has the potential to deepen the reflection and potentially the learning.^19^^,^^20^ Mentored reflective practice, faculty role-modeling, and formative feedback can promote professional development.^23^

RW has previously been used in global health education to assess predefined global health competencies and to assess those competencies identified by the Accreditation Council for Graduate Medical Education (ACGME).^15^^,^^24^^,^^25^ RW has also been used to determine whether trainees are meeting the expected educational and clinical objectives during global health training.^26^^–^^28^ In these previous applications, RW was conducted in an open-ended manner with participants writing about an impactful clinical scenario rather than responding to a prompt to elicit and demonstrate specific learning objectives.

The aim of our study was to understand whether prompted RW during GCRs could provide a means to assess medical student learning while participating in GCRs. Ours is the first study that has used RW as a means to assess AAMC competency domains during GCRs.^29^ We hypothesized that GCRs would offer a rich learning environment to develop cross-cultural awareness and humanism, given that these competencies have been well-documented during GCRs.^10^^,^^15^^,^^30^^,^^31^ We also anticipated that a prompt on resources would elicit reflections comparing health systems, thus demonstrating an understanding of different systems-based practices, one of the AAMC competency domains. Through prompted RW during GCRs, we were surprised to learn that medical students are able to reflect on and demonstrate learning in all the AAMC competency domains, which makes RW useful as an assessment tool.

## METHODS

### RW prompts.

GHP faculty developed the RW prompts to enhance learning objectives during GCRs. The first RW prompt, assigned before a mandatory predeparture orientation, is a reflection on ethical challenges that can occur when engaging in short-term global health learning experiences. This prompt was created after several years of engaging with students on the ideas addressed in a short film on the ethics of GCRs. The additional three prompts were chosen based on common themes that emerged during the debrief meetings that followed the global health rotations. Students received the three remaining prompts at the beginning of each week of their GCR, with encouragement to reflect on the prompt throughout the week and respond with a written reflection by the end of the week. Peers who were participating in the GCRs across locations and GHP faculty engaged interactively with the students by commenting on their online submissions to the RW prompts. For the purpose of this study, we analyzed those prompts that were assigned across most or all 5 years of the DGSOM GHP’s GCR, including “Ethics of Wanting to See,” “Learning Lessons,” “Resources,” and “Cultural Humility,” as shown in Table 1.

**Table 1:** Reflective writing prompts

RW title (and timing of assignment)	RW prompt	Years RW prompt used
Ethics of Wanting to See (before predeparture orientation)	Students watched the film *First Do No Harm: A Qualitative Research Documentary*^39^ and then responded to the prompt: “Is there anything wrong with wanting to “see”? Is this different from pre-clerkship observation, or medical students being excited about traumas they have seen or babies they have delivered at their home institutions? Does “voyeurism” only apply when away from home?”	2014, 2015, 2016, 2017, 2018
Learning Lessons (end of first week of GCR)	“What was the most surprising thing that happened to you this week? This could be in the clinical setting (such as an interesting clinical case) or adjusting to your new environment.”	2014, 2015, 2016, 2017, 2018
Resources (end of second week of GCR)	“Ask a provider if there was one tool or resource they wish they had available in their clinic or hospital, and how they compensate without having this tool or resource. Be careful to communicate that you are not promising to provide this tool but rather you are interested in how care is provided when resources are scarce. AND/OR Interview a patient about the economic burden of a hospital or clinical visit. Is there health insurance? How do patients pay for their medical care? Does this include the cost of medications? What is the burden of this visit on their salary or savings?”	2014, 2016, 2017, 2018
Cultural Humility (end of third week, or shortly after completing GCR)	“There is a cultural barrier between you and the patients you are seeing, but there may also be cultural, language, religious, socioeconomic, or other barriers between the medical staff and the patients. Can you describe how cultural humility is practiced by the providers you are working with? Can you share an anecdote or example of cultural barriers within the community you are working? Have you seen examples of cultural sensitivity or cultural insensitivity that will influence your own practice?”	2014, 2015, 2016, 2017, 2018

GCR = global clinical rotations; RW = reflective writing.

### Data analysis.

We conducted qualitative thematic analysis of the students’ writing from 2014 to 2018. Written reflections were deidentified and grounded theory methodology was used to develop themes through an iterative process.^32^ Our codes were initially open and inductive until we moved toward our final set of central themes, as they emerged from the writing during our analysis. Two of the researchers (T. W., P. P.) coded the 2014 and 2015 reflective writing assignments manually and met weekly to compare coded themes. Using a constant comparative method, we found that the themes that emerged during the iterative process corresponded to knowledge and skills described within each of the eight AAMC competencies. When coding an utterance with different themes, T. W. and P. P. discussed differences until consensus was reached. The codebook was created and refined based on the data set from 2014 and 2015, which was 30.5% of the total data set (see Table 2). Both positive and negative examples of the competencies addressed in the blogs were recorded. For example, if a student noted observing a lack of professionalism, this was counted as learning attached to the competency on professionalism. When the same competency was addressed multiple times in the same paragraph, it was counted only once unless it was a different example of the same competency. The themes outlined in the codebook developed from the 2014 and 2015 data were subsequently used by T. W. to code the reflective writing assignments for all 5 years using NVivo software. G. A. and H. M. also coded the entire data set using NVivo software and consulted with T. W. on divergences in coded themes. Approval for this study was provided by the UCLA Institutional Review Board.

**Table 2:** The codebook with AAMC General Physician Competency Domains corresponding to the themes described in RW

AAMC General Physician Competency Domains	Description of RW themes*
Patient Care	Caring for patients
Knowledge for Practice	Psychosocial and cultural influences on health
Practice-Based Learning and Improvement	Learning, including knowing one’s limits, gaps in knowledge, skills or attitudes, ideas for improving quality of practice, understanding communities to improve care
Interpersonal and Communication Skills	Communication with both colleagues and patients across socioeconomic and cultural backgrounds, including language barriers
Professionalism	Respect for patient privacy and autonomy, and sensitivity and responsiveness to a diverse population
Systems-Based Practice	Description of various healthcare delivery settings and systems relevant to one’s clinical specialty and skills, including cost-awareness and risk-benefit analysis, often involving comparing the U.S. system with the host system
Interprofessional Collaboration	Being a team player with respect for colleagues and ethical integrity. In a global health setting, this also includes respect for host mentors and their knowledge and expertise
Personal and Professional Development	Self-awareness of knowledge, skills and emotional limitations, while remaining mature and adaptable in an ambiguous and often changing clinical environment

AAMC = Association of American Medical Colleges; RW = reflective writing.

* When these themes were described in RW, they were coded to the corresponding AAMC Competency Domain.

## RESULTS

A total of 135 fourth-year medical students responded to at least one of the four RW prompts between 2014 and 2018, with between 106 and 135 written responses to each prompt, resulting in a retrospective review of 487 individual responses to writing prompts (see Table 3).

**Table 3:** Number of students responding to reflective writing prompts

	2014	2015	2016	2017	2018	Totals
Ethics of Wanting to See	20	22	23	41	29	135
Learning Lessons	9	23	23	39	26	120
Resources	14	n/a	23	40	29	106
Cultural Humility	16	21	22	39	28	126

The themes that were uncovered during data analysis were closely tied to the eight AAMC competencies: Patient Care (PC), Knowledge for Practice (KP), Practice-Based Learning and Improvement (PBLI), Interpersonal and Communication Skills (ICS), Professionalism (P), Systems-Based Practice (SBP), Interprofessional Collaboration (IC), and Personal and Professional Development (PPD). We found evidence of all the competencies in the students’ written responses to each of the four prompts, although reflections on learning looked different for each of them, as outlined below.

### Prompt 1 (predeparture): Ethics of wanting to see.

Although all eight AAMC competencies were coded in response to the Ethics of Wanting to See prompt, which was assigned before beginning the GCR, PPD, and PBLI, were the competencies that most commonly emerged from RW in response to this prompt (Table 4).

**Table 4:** The number of times each competency was coded in reference to each of the four reflective writing prompts

	PC	KP	PBLI	ICS	P	SBP	IC	PPD
Ethics of Wanting to See	6	13	51	6	42	9	25	62
Learning Lessons	47	17	22	9	13	99	39	15
Resources	27	13	14	2	4	103	25	5
Cultural Humility	19	42	70	68	57	61	40	8

PC = patient care; KP = knowledge for practice; PBLI = practice-based learning and improvement; ICS = interpersonal and communication skills; P = professionalism; SBP = systems-based practice; IC = interprofessional collaboration; PPD = personal and professional development.

#### Personal and professional development.

In response to the Ethics of Wanting to See prompt, the students reflected on their anticipated role within in the medical team while participating in GCRs.

Students often expressed an awareness of their lack of experience. One student explained, “The more I learn and experience throughout training in medicine, it is humbling to realize how ill-equipped I really am to help others.” Another student expressed particular awareness of their training level: “Seeing an American wearing a white coat might—in the eyes of local staff and patients—extend[s] my privileges beyond what they should be, but it is my job to police myself carefully over what aspects of clinical duties I am qualified to participate [in].” Similarly, another student explained that it is not the role of students to try to assert the superiority of their practice in other countries: “When seeing patients or rounding with the teams here, my intention is to learn how medicine is practiced [here], and not to offer my own expertise. … I appreciate the local attendings’ curiosity about how treatment algorithms might be slightly different, but we are in no position to offer superior medical practices to those that already exist in that country.”

Students also expressed an acknowledgment of receiving more learning than they could contribute, and the hope of being able to give back to future patients. “We have opportunities to learn as much as we can about patients and the medical world around us, so that we can help future patients and healthcare systems. I agree that we should always be compassionate and aware of our roles as well as the ethical burden of being a voyeur, with the ethical contract in mind that we will learn from these experiences and do good for others in our future careers.”

#### Problem-based learning and improvement.

In describing motivations for participating in GCRs, one student explained, “I plan to work with the underserved, underresourced in the future, and the skill sets I aim to build are ones that will give me the tenacity to navigate the health system, the awareness of the difficulties/disparities faced by varying populations, and the language to address those issues appropriately and respectfully.” Many students felt that being in a different clinical setting, diverse from their medical school experience, would provide the opportunity to acquire knowledge about diseases not seen in their home institution and an opportunity for self-reflection when faced with a new cultural environment. One student described, “I hope to improve my cultural competency, to see medicine in a different context, to learn about a plethora of illnesses and diseases not readily seen in the States, and to become a more adaptable and skilled version of myself.” Another student wrote, “I think that in contemplating a career focused on addressing healthcare disparities and health system inadequacies globally and locally, on-the-ground experience is paramount. … This type of knowledge is often very specific to a region or community, and as such must be learned in that setting.”

### Prompt 2 (week 1): Learning lessons.

Although all competencies were recorded, as shown in Table 4, the three most highly referenced competencies coded in response to this prompt were SBP, PC, and IC.

#### Systems-based practice.

One student explained the relative importance of the physical exam, stating that “There is a big premium [here] put on physical exam and clinical judgment, as labs take 8 hours and a stat CT is about the same.”

Another student alluded to the role family members noting, “It’s almost required to bring a family member to appointments, especially anything regarding surgery because typically consent forms are signed by a family member and not the patient themselves, even if the patient is capable and fully informed and of right mind. It reminded me of an ethics question posed to us in doctoring about patient autonomy versus family wishes to keep certain aspects of the patient’s diagnosis/prognosis vague. In the US, patient autonomy is highly regarded and it was pretty much agreed on that as a physician, our priority is first and foremost our patient and their wishes, and only if they agreed to be withheld information would we acquiesce to family requests to keep the patient in the dark about their own disease process.”

Some students reported on the similarities between health systems. “Despite some differences between the structure of … training, I am more amazed by the striking similarities—finding the elusive consultants, trying to keep orders and laboratory results organized, and waking up the post-call intern during noon conference. As an outside observer, I struggled to understand the norms around privacy, patient-involvement in medical decisions, physician-patient rapport, and physician hierarchy. However, these anecdotes easily could have taken place back home.”

Other students described differences in systems-based practices, which gave them a new appreciation for their home institution. “While perhaps not surprising, the differences in healthcare delivery are striking and extremely interesting. The difference in patient flow in the ED [emergency department] due to limited resources is immediately obvious. The support staff is sparse and their level of training is quite limited when compared to US standards. I have developed a new perspective (in addition to gratitude for the support we have back home) on things I usually take for granted including blood draws, IV lines, intake and discharge paperwork, etc.”

#### Patient care.

Students often described medical conditions previously never seen or seen only in textbooks. In describing patient care, students also reflected on caring for patients within resource limitations: “One patient presented with lower extremity paralysis and was suspected of having some active spinal process. However, he was unable to afford a CT scan, and so we had to treat him for the most likely diagnosis.”

Similarly, another student identified a complex case in which limited resources impacted the patient care provided. “One interesting case I had so far was a child with acute lymphoblastic leukemia (ALL) s/p chemotherapy who was referred to the … hospital for management of fever and neutropenia. During the hospitalization, [the] patient developed painful facial swelling and purplish skin discoloration without obvious neurologic changes, concerning for facial cellulitis. Basic infectious workup was sent and a head CT with contrast was performed, which showed microabscesses in the parotid glands bilaterally. [The] request for MRI to better characterize the lesions were declined due to cost restriction.”

#### Interprofessional collaboration.

IC was coded when examples of respect for colleagues and ethical integrity were described. One student wrote, “It is humbling and inspiring to see these physicians practice evidence-based care and still be very cost-conscious and rational.” The students regularly reported how grateful they were for the teaching they received from their hosts, sharing “I’m constantly reminded about how much I never knew or might have forgotten over the past few years, and we have benefitted from some very patient and generous teachers.” The students’ respect for host-site providers was extended further when they witnessed effective teamwork despite challenges placed by both the health system and limited resources. One student described this dynamic as “[Here is] a patient who had a stroke a day ago, and the whole multidisciplinary team is ecstatic that she was successfully scheduled for a MRI in 4 months. The staff here have an energy and passion for what they are doing that is truly admirable. They work tirelessly to make the best out of what they have, and to push each resource to its limits. And when the system they work in is doing everything it can to prevent them from providing quality care, they just work through it with a smile. There is so much to learn from these health workers.”

### Prompt 3 (week 2): Resources.

Although all competencies were coded, SBP was the most prominent competency coded within the responses to this prompt, as shown in Table 4. It is worth noting that the other competencies were significantly less common, which is important to our discussion.

#### Systems-based practice.

SBP references included comparisons between healthcare systems in the United States and the host site, including resources used in patient care, the roles of medical professionals, and the structure of medical education. The responses to the Resources prompt tended to be descriptive and were mostly focused on the costs associated with medical care, including opportunity costs such as time and transportation. The responses were also centered on the resources that were lacking, rather than ways of providing quality care in the absence of resources. One student explained that host-site physicians “feel they need to tailor their treatments to what patients can afford. Although this type of decision regarding care may be more feasible in an outpatient setting, in the emergency room the attending felt it can dictate a viable vs. fatal outcome.” In addition to needing to tailor treatment to what patients could afford, the students also addressed the lack of access to diagnostic tests. One student argued that “not having the CT scan, the ECHO [echocardiogram], the blood bank, and the labs really does make a difference. The patients here tread through huge lines, travel vast distances, and wait months to complete a workup that would take days in the USA. They suffer with worsening symptoms and terrible pain as they await treatment. Their diseases slowly progress to advanced stages we rarely see in the United States, and unfortunately, even with all the resources in the world, there is little to do to help patients at this point.”

### Prompt 4 (week 3): Cultural humility.

This prompt had the most coded competencies of all the reflective writing prompts, touching heavily on most of the AAMC competencies, including PBLI, ICS, P, KP, and PPD, as shown in Table 4.

#### Problem-based learning and improvement.

Many students described an increased awareness of the costs of medical care based on learning during their GCRs; for example: “It has been less than a week, and this experience has already solidified my desire to really strive for cost-effective care in my future practice.” Others became more aware of the role that family plays in medical decisions across cultures, describing that “My experiences working on the pediatrics ID [infectious disease] service certainly have made me think about my own practice in the future. Not only do I have the pediatric patient in mind but also the parents and other family members. To be culturally sensitive, I will have to understand what the family dynamics are. For a pediatric patient on viral precaution from vertical transmission, we have to respect the mother’s wishes here to uphold what she deems important to her family—to keep her HIV status and route of transmission secret to the child and father.”

Other students provided examples of their desire to understand communities to improve care in their future practice, “This will help me because now I know a lot more remedies that are popular in [location of the GCR] and will ask my patients about them and won’t be opposed to respecting that practice if my patient wants to do that. Hopefully they’ll feel more comfortable sharing their practices with me.” Students also reported an increased appreciation for nonallopathic health practices, sharing “What I am now further convinced of is that I need to more open-minded, aware about, and actively seek out the different views of and approaches to health. Even though I don’t quite yet understand Traditional Chinese Medicine’s intricacies and will not be able to use Western medicine–colored glasses to rationalize its techniques, I’m glad that these treatments that stemmed from experience and tradition were able to take care of this patient.”

For those students participating in GCRs where they do not understand the language, they reflected on language practices in healthcare. “The experience has influenced me to be extra motivated to seek out translators and ensure that I am able to properly communicate with my patients—regardless of how swamped I may be or how tempted I may be to take shortcuts and forgo a translator.”

#### Interpersonal and communication skills.

Students provided both positive and negative examples of ICS in response to the Cultural Humility prompt. “The fact that he (U.S. trained physician working in-country full-time as host-site faculty) is able to speak to these patients in [the patient’s language] definitely allows him to form a connection with his patients that I don’t think many other outside doctors are able to do. … It was always funny to see the mom’s responses when this American doctor started speaking [the local language]—they all instantly started laughing. I could tell it made the patients feel a lot more comfortable and I would bet that his patient compliance rates are higher because of it.” Another student noted: “There were some instances where the paternalistic side of medicine came through and patients were often ‘told’ how care was going to be provided. At times, the patients’ input was disregarded as the care team felt they had poor medical insight.”

A barrier in language sometimes led to students’ awareness of nonverbal communication, noting, “While the language barrier limited my verbal communication with patients, it triggered my attention to the nonverbal communication displayed by patients.”

#### Professionalism.

One student wrote, “The providers are very conscious of cultural taboos specific to their community, and routinely point out specific examples to us students. For instance, leprosy has a lot of associated stigma, so they instead call it Hansen’s Disease in order to protect their patient’s privacy.” The students sometimes described a lack of professionalism during their rotations, such as an incident involving “two White paramedics and a Black patient who spoke no English or Afrikaans and was being brought in after getting hit by a car. … The medics were ridiculing the patient’s screams, giving him commands in English knowing that he could not understand, and ridiculing him further for not listening. This was witnessed by multiple staff members and physicians who responded with indifference from some and joining in on the paramedics’ jokes from others.”

#### Knowledge for practice.

In response to the Cultural Humility prompt, students described cultural practices that influenced patient health. For example, many wrote about coming into contact with patients seeking medical attention after treatment from a traditional healer. One student wrote, “Then there are the many, many kids presenting with acute gastroenteritis due to the traditional medicine they receive. Apparently, there is a local belief that when thunder makes a baby cry, it’s not due to sensory stimulation, but due to the gaseous storm in the baby’s stomach caused by the thunder. And the solution? Herbal enemas.” Another student was mentored by the host-site provider to spend more time taking a social history, to develop a deeper understanding of the psychosocial or cultural influences on health. The student explained, “We sent one patient to adherence counseling because she came back for her ART [antiretroviral therapy] refill 2 weeks early after running out of pills. After her counseling session, we learned that she was in fact taking her pills. However, her husband who lives with his second wife steals her pills, either for himself or for his other wife.”

#### Personal and professional development.

The Cultural Humility prompt, offered at the end of GCRs, also provided an opportunity for students to reflect on the entirety of their GCR and consider how they have been transformed by their experience. As one student described, “I think it is the times when we are uncomfortable and confronted with difficult situations that we can become more aware of ourselves and tendencies. The things we take for granted and don’t appreciate enough.”

## DISCUSSION

Our study sought to investigate whether prompted reflective writing could capture the extent and quality of learning that occurs during medical student participation in global clinical rotations (GCRs). Through a qualitative analysis of medical student responses to RW prompts during GCRs, we found that students displayed learning related to all eight AAMC competencies expected of all physician trainees. We predicted that GCRs would expose students to new health systems and expected that the AAMC competency domain of SBP would be the most commonly referenced competency, which was confirmed by our qualitative analysis. However, we also found that prompted RW during GCRs allowed for the demonstration of learning in all eight AAMC competencies, including the competencies of PPD, KP, IC, and PC. In demonstrating practice-based learning and improvement, students also reflected on how the experiences during their GCRs might impact their future careers as physicians.

It is possible that our medical students were primed to reflect thoughtfully and critically on their learning before participating in the GCR, despite not having been taught how to do reflective writing per se, given that the first prompt, Ethics of Wanting to See, was assigned to accompany the predeparture training. We recognize that reflecting critically and vulnerably on one’s experience is a learned skill, and we intentionally gave our students the opportunity to engage in prompted RW and to see reflections from their peers and responses from faculty before the GCR. This prompt resulted in students reflecting largely on their reasons for wanting to engage in GCRs and thereby demonstrating competencies of personal and professional development, problem-based learning and improvement, and professionalism. In considering their goals and objectives for participating in the GCRs, students expressed that these experiences would allow them to better care for future patients, both at their home institution and globally.

The Learning Lessons prompt provided the greatest opportunity for students to reflect on systems-based practice while also deeply engaging with the patient care and interprofessional collaboration competencies. In contrast, the Resources prompt resulted in students reporting on the lack of resources in healthcare and on the economic burdens to patients. This resulted in the dominant theme of SBP in response to this prompt and much less reflection on the other competencies. The wording of the Resources prompt may have invited students to approach their host site from a deficit perspective rather than potentially sharing more about strengths and innovation found in the host’s health system. In asking students to reflect on resources that were missing or economic burdens in healthcare, they were not positioned to engage in critical reflection, which may have contributed to less diversity in the competencies unveiled in response to this prompt.

The Cultural Humility prompt had the most coded competencies, with all eight competencies identified and six of the eight heavily represented. This prompt, perhaps due to the timing of the prompt, which was assigned toward the end of the elective, allowed students to demonstrate ways in which GCRs changed or transformed their thinking around their own practice of medicine. In considering how the experience of the GCR would benefit future patients, prompted RW in GCRs might offer students the opportunity to demonstrate transformative learning; the idea that learners facing new perspectives do not merely apply their old understanding to new situations but rather, through critical reflection, develop a new understanding.^33^^–^^36^ Because GCRs are often offered to medical students in their final year of medical school, guided reflection during this time provides an opportunity to reflect on both past learning and future practices.

Engaging with students on GCRs through the prompted RW online blog allowed UCLA DGSOM GHP faculty to have further insight into the student learning experience despite not being physically present during the GCR. Given that global health learning often happens through GCRs without home institution faculty present, it may not be possible to use typical tools to assess and provide feedback to learners. Additionally, host-site faculty may not be familiar with the students’ medical education curricula, assessment process, and learning objectives for rotating medical students and may not have time or resources to conduct learner assessment. RW allowed the GHP faculty to hear from the students as they are learning, reflecting, and transforming during their GCR and provided the opportunity for formative feedback to be incorporated during the GCR without further burdening host-site faculty.

Although prompted RW during GCRs showed that students were displaying learning consistent with AAMC competency domains expected of all physicians, it is important to note that certain measures needed to be in place for the UCLA DGSOM to create effective learning experiences for students. First, this program required student participation in a structured predeparture orientation with an emphasis on professionalism, ethical engagement, and cultural humility during their GCRs.^6^ Secondly, student learning was likely enhanced by sending students to host sites with strong interinstitutional partnerships, which resulted in active supervision and mentorship by host-site faculty. Where possible, DGSOM UCLA partners are invited to engage in the reciprocal exchange of students, postgraduate trainees, and faculty. Within these strong partnerships, it is plausible that the host-site faculty felt more supported and invested in student learning across competency domains.^37^^,^^38^

### Study limitations and future research.

Our RW data suggest that medical students demonstrate AAMC competencies while participating in GCRs, but we cannot be certain whether these competencies were acquired through learning experiences within or outside GCRs or to what extent the process of writing and interacting with peers and faculty on blogs facilitated the acquisition of competencies. Future research could include asking medical students participating in domestic clinical rotations to engage in prompted RW to identify any differences in learning between the two groups. In the absence of a direct comparative study, we cannot establish causality between GCRs and the attainment of competencies. Another limitation of the study is that our colleagues who supervise students during GCRs at the host sites did not have the opportunity to read and respond to students’ RW. Not only might these interactions have changed the data, but they might also have provided our partners with opportunities to share their experiences with hosting and teaching our students. We also understand that the prompts assigned were closely aligned with medical school learning objectives and that more open-ended reflection might have achieved different results. Future work might include a comparison of competencies demonstrated by students participating in GCRs in different low- and middle-income settings, in addition to incorporating a control group engaging in RW while participating in a clinical rotation in an underserved setting in the United States. We could also consider incorporating focus groups to allow students to respond to specific content shared in the RW to develop a deeper understanding of how GCRs affect learning. Additionally, future studies may benefit from collecting follow-up data from students who had participated in GCRs to assess whether they incorporated changes in practice or approaches to medicine as a result of their GCRs or whether the competencies demonstrated during the GCRs were sustained over time.

## CONCLUSION

GCRs have previously been shown to provide medical students with rich learning experiences. The qualitative data from this study illustrate that prompted RW during GCRs can be an opportunity for faculty to understand to what extent students are achieving AAMC competencies. Given the potential of GCRs to contribute to and enhance student learning across all physician competencies, we recommend further research into additional ways to assess student learning during GCRs, with a focus on learning that is unique to GCRs, as well as opportunities for transformative learning during GCRs.
